# Evolutionary Origins of Metabolic Reprogramming in Cancer

**DOI:** 10.3390/ijms232012063

**Published:** 2022-10-11

**Authors:** Natalia García-Sancha, Roberto Corchado-Cobos, Aurora Gómez-Vecino, Alejandro Jiménez-Navas, Manuel Jesús Pérez-Baena, Adrián Blanco-Gómez, Marina Holgado-Madruga, Jian-Hua Mao, Javier Cañueto, Sonia Castillo-Lluva, Marina Mendiburu-Eliçabe, Jesús Pérez-Losada

**Affiliations:** 1 Instituto de Biología Molecular y Celular del Cáncer (IBMCC-CIC), Universidad de Salamanca/CSIC, 37007 Salamanca, Spain; 2 Instituto de Investigación Biosanitaria de Salamanca (IBSAL), 37007 Salamanca, Spain; 3 Departamento de Fisiología y Farmacología, Universidad de Salamanca, 37007 Salamanca, Spain; 4 Instituto de Neurociencias de Castilla y León (INCyL), 37007 Salamanca, Spain; 5 Lawrence Berkeley National Laboratory, Biological Systems and Engineering Division, Berkeley, CA 94720, USA; 6 Berkeley Biomedical Data Science Center, Lawrence Berkeley National Laboratory, Berkeley, CA 94720, USA; 7 Departamento de Dermatología, Hospital Universitario de Salamanca, Paseo de San Vicente 58-182, 37007 Salamanca, Spain; 8 Departamento de Bioquímica y Biología Molecular, Facultad de Ciencias Químicas, Universidad Complutense, 28040 Madrid, Spain; 9 Instituto de Investigaciones Sanitarias San Carlos (IdISSC), 28040 Madrid, Spain

**Keywords:** cancer, tissue repair, inflammation, regenerative phase, evolution, heritability, intermediate phenotypes

## Abstract

Metabolic changes that facilitate tumor growth are one of the hallmarks of cancer. These changes are not specific to tumors but also take place during the physiological growth of tissues. Indeed, the cellular and tissue mechanisms present in the tumor have their physiological counterpart in the repair of tissue lesions and wound healing. These molecular mechanisms have been acquired during metazoan evolution, first to eliminate the infection of the tissue injury, then to enter an effective regenerative phase. Cancer itself could be considered a phenomenon of antagonistic pleiotropy of the genes involved in effective tissue repair. Cancer and tissue repair are complex traits that share many intermediate phenotypes at the molecular, cellular, and tissue levels, and all of these are integrated within a Systems Biology structure. Complex traits are influenced by a multitude of common genes, each with a weak effect. This polygenic component of complex traits is mainly unknown and so makes up part of the missing heritability. Here, we try to integrate these different perspectives from the point of view of the metabolic changes observed in cancer.

## 1. Introduction

Cancer is associated with a series of metabolic changes initially thought to result from tumor behavior. However, they were subsequently shown to contribute to its pathogenesis. Indeed, the metabolic changes observed in the tumor are now considered one of the hallmarks of cancer [1]. These metabolic changes result from molecular and cellular interactions between the tumor cells and the stromal compartments and allow tumor growth [2]. The de novo metabolic activity in the stroma is triggered by the tumor cells after activating oncogenes and the loss of suppressor genes. This signaling causes the stroma to be functionally activated, thereby allowing tumor growth [3].

We may wonder how these molecular and cellular metabolic mechanisms that permit tumor growth originated. A possible explanation comes from the fact that these cellular and molecular metabolic changes are usually already present in the organism’s physiology. Indeed, all of them have their physiological correlate in the process of physiological tissue growth and, more specifically, in the wound repair phase [4]. Certainly, Dvorak had considered cancer a wound whose healing process never finished [5]. In short, a tumor is a chronic reparative phase, and metastasis is the maintenance of that same chronic reparative phase at distant locations. Moreover, globally, the exact molecular and cellular metabolic processes driving tumor growth also occur in the chronic reparative phase of normal tissues [4]. The presence of pretty similar metabolic interactions in tumor growth and in the tissue repair process sheds light on how these metabolic interactions must have been constructed and improved over time. Every biological process must be justified from an evolutionary perspective. The explanation of diseases and their prevalence cannot escape the ramifications of this brilliant intuition ascribed to Dobzhansky [6].

Indeed, these molecular and cellular metabolic interactions might be formed, each with their own peculiarities, during the evolution of species in each phylum. Since the appearance of complex life forms, there has been strong selection pressure to meet the need to regenerate tissues after environmental aggressions. This environmental selection pressure must have generated the molecular processes and cellular metabolic interactions that allow tissue repair, which eventually enable cancer to grow.

These molecular and cellular metabolic mechanisms permit wound repair and tumor growth. The growth of normal and pathological tissues are complex traits, which are the consequence of many intermediate molecular and cellular phenotypes controlled by many genes [7]. However, one of the characteristics of complex traits is that the phenotypic variation due to genetics (known as heritability) is only a tiny percentage of what is estimated (known as expected heritability) because the genes responsible for it are not found. This lack of localization of the genes is known as missing heritability [8]. We have proposed that missing heritability originates in many genetic variants that contribute to intermediate phenotypes. It is possible to find such genetic variants associated with intermediate phenotypes of a complex trait. However, their contribution to the central complex phenotype is not strong enough to be detected in association with it [7].

This review explores the similarities of these molecular and cellular metabolic interactions with those in the tissue repair process. We explain how these metabolic interactions must have been assembled throughout evolution to achieve efficient tissue repair. Finally, we discuss how intermediate metabolic phenotypes contribute to phenotypic variation between individuals in tissue repair and cancer development. We conclude by considering what implications this architecture of metabolic interactions has for locating genetic variants that explain tumor susceptibility

## 2. Towards Physiological Integration: Tumor Growth Shares Metabolic Mechanisms with the Injury Repair Process

The fundamental nature of the tumoral process is that it consists of the growth of a tissue. Tissues also grow under normal physiological conditions, but only in response to the organism′s physiological needs, such as for wound healing, leukocyte production after infections, or repairing bone fractures, but once the physiological objective has been met, the process ceases. Cancer, however, is a tissue that grows uncontrollably, is not driven by the organism′s physiological needs, and is not self-limited. In fact, it has been proposed that cancer resembles the process of tissue injury repair [5].

The repair of tissue injuries has four phases. The first phase, the hemostasis phase, aims to prevent death through bleeding, in which platelets and the coagulation system are essential. The second phase, which aims to prevent infections, is the inflammatory phase, which features considerable immune system activity against pathogens and in which the remains of necrotic tissues are eliminated. Microorganisms and necrotic tissues present proinflammatory signals to the immune system, and their elimination allows the subsequent reparative stage to take over. The first two phases ultimately aim to prevent death from bleeding or infections. In the regenerative or reparative phase, during which the new tissue is formed, the tissue parenchyma proliferates. The activity of the surrounding stroma is vital in this process. The final phase is tissue remodeling, which aims to prevent excess fibrous tissue from forming, which would lead to defects in function. This four-stage process occurs in superficial wounds and internal tissue injuries.

The tumor parenchyma recruits and activates the stroma to grow in the same way normal parenchyma does in repairing tissue injuries. In other words, tumor growth mimics the development of normal parenchyma during the regenerative phase of physiological tissue repair.

### 2.1. An Antiinflammatory Immune Response Prevails in the Physiological Regenerative Phase and in Tumors

As in normal tissue growth, tumor proliferation is coordinated with immune and endothelial cells [9,10], the stromal microenvironment being very similar in both cases. The metabolites released by proliferating parenchymal cells have a paracrine effect, leading to the activation of stromal fibroblasts and functional changes in immune [11,12] and endothelial [13,14] cells.

The inflammatory response phase is initiated by the innate immune system [15]. Blood monocytes arrive at the site of injury attracted by molecules that are released from the broken cells of the necrotic parenchyma. These molecules are collectively known as alarmins or DAMPs (Damage-Associated Molecular Patterns). In general, they are small metabolites such as ATP (Adenosine Triphosphate), uric acid, and nucleotides [16,17,18,19]. Monocytes at the injury site differentiate into macrophages. Macrophages are essential cells in the previous inflammatory phase and the second or proper reparative phase. Macrophages that potentiate inflammation are in an M1 functional state. M1 macrophages synthesize a large amount of nitric oxide from arginine. Macrophages participating in the reparative phase are in an M2 functional state, where arginine would preferably be transformed into ornithine. M1-type macrophages, with proinflammatory activity, eliminate necrotic tissue and microorganisms that may be contaminating the wound [20,21,22,23]. Indeed, the response to a wound begins with an inflammatory response based on neutrophils and M1 macrophages, which are activated by IFN-γ (Interferon-γ)/TLRs (Toll-Like Receptors) to avoid microbial contamination. It has been suggested that M1-macrophage activity is unnecessary if no microbes are present [24,25], so its role is to control infection rather than be involved in the repair process itself. Indeed, complete wound repair can only occur when the inflammatory phase ends [26,27].

Once the necrotic remnants have been removed, the inflammatory phase ceases, and the regenerative phase begins. Macrophages acquire a functional M2, immunosuppressive and regenerative phenotype facilitated by environmental metabolites and T lymphocytes. M2 macrophages fulfill four physiological functions. First, they end the inflammatory phase through their suppressive activity. Second, they activate parenchymal cells to initiate regeneration [20,28,29]. Third, they release chemoattractant factors that recruit fibroblasts to the wound. In fact, as in wound-healing processes, stromal cells are recruited to the tumors by chemotaxis [30]. Finally, they functionally activate fibroblasts, which requires great functional capacity. From a morphological point of view, this manifests as the differentiation to myofibroblasts [31,32,33], secretion of extracellular matrix (ECM) components, and the synthesis of αSMA (α-Smooth Muscle Actin) [34,35,36,37]. Molecules that induce myofibroblast differentiation include TGFβ (Transforming Growth Factor β), PDGF (Platelet-derived Growth Factor), radical oxygen species (ROS), and ECM mechanical forces.

The tumor stroma has many characteristics similar to those observed in the regenerative phase of wound repair, and tumors can even be described as having a continuous aberrant regenerative response. Since it is a regenerative-like phase, the predominant cells are not those of the proinflammatory immune system but rather the immunosuppressive cells that resolve inflammation and facilitate regeneration. These immunosuppressive cells encompass macrophages with the M2 phenotype, regulatory T cells, and myeloid suppressor cells [38,39,40,41].

T helper cells (TH) contain and release cytokines with essential functions in the tissue repair process. The T helper 2 (TH2) lymphocyte response produces molecules that have both anti-inflammatory and regenerative activity. These include TGFβ, which suppresses the inflammatory response and is also a potent profibrotic. TH2 cells produce 12/15-Lipoxygenase and TREM-2 (Triggering Receptor Expressed on Myeloid cells 2), which are anti-inflammatory mediators that enhance wound repair [24,42,43]. Thus, a complete repair can only occur if the inflammatory phase has concluded [26,27].

Once the T helper 1 (TH1) lymphocyte activity has eliminated the infection, it is inhibited by the TH2 response. At the same time, the action of TH2 induces the production of a granulation tissue that replaces the fibrin clot, whose function is to plug the lesion to prevent more microorganisms from entering rapidly. This granulation tissue is made up of type III collagen produced by myofibroblasts. This collagen is structurally weak but can be synthesized quickly. It is then replaced by the stronger type I collagen. Myofibroblasts produce both types of collagen in response to the IL-4 (Interleukin-4) and IL-13 produced by TH2. TH2 cytokines also induce the expression of molecules, such as arginase, MMP12 (Matrix Metalloproteinase-12), and TREM-2, that directly or indirectly participate in tissue repair [44]. The main target of IL13 is macrophages that are transformed into having an M2 phenotype [45].

### 2.2. The Metabolic Reprogramming in Cancer

One of the hallmarks of cancer is the metabolic changes, which aim to permit tumor growth [1]. The triggers of these metabolic changes are in the tumor parenchymal cells, in which oncogenic mutations induce an imperative need to proliferate and cause tumor initiation and progression. Cancer cells undergo significant metabolic reorganization during disease progression, tailored to their energy demands and fluctuating environmental conditions. Indeed, a series of metabolic changes occur during tumor development that favors its growth, including (i) increased glycolysis, decreased activity of the Krebs cycle, and acidification of the interstitium due to the release of lactate [46,47]. (ii) Cancer cells show increased glutamine and other amino acid utilization [48]. (iii) Tumor cells capture large amounts of fatty acids and synthesize complex lipids to construct cell membranes [49]. (iv) Tumor cells adapt to a chronic deficit of nutrients and oxygen in the interstitium. Our group has recently reviewed these and other properties in detail [2]; we refer to that and other current reviews on the subject [50,51,52].

Indeed, proliferating tumor cells have high greed for glucose and amino acids. This fact leads to a relative deficit of both in the tumor interstitium. In addition, pyruvate enters the tricarboxylic acid cycle with difficulty and is mainly transformed into pyruvate (Warburg effect) [53]. Pyruvate is released into the interstitium and contributes to its acidification. These metabolic changes result from the interaction between tumor cells and stromal myofibroblasts. The metabolic changes in tumor cells include protein anabolism and the synthesis of cell membranes and nucleic acids, permitting cell proliferation. These changes are linked to catabolism and autophagy in stromal myofibroblasts to release nutrients for the cells of the tumor parenchyma. Metabolic changes lead to an interstitium deficiency in nutrients, such as glucose and amino acids, and acidification by lactic acid [2,50,51,52].

Initially, the Warburg effect was attributed to the entire tumor, specifically cancer cells. However, many tumors have an important stromal component, and a more detailed study proposed that in many cases, the Warburg effect would not be present in the tumor cells but in the stroma. This model has been called the reverse Warburg effect or coupling model. According to this model, a symbiosis would be established between stromal and tumor cells that would allow the growth of both cell populations [54]. The coupling model proposes integrating the various metabolic changes observed in cancer more functionally. This model is based on the premise that most human tumors, such as the breast, stomach, and pancreas, contain considerable stroma. The Warburg effect would preferentially occur in the predominant stromal cell type, i.e., cancer-activated fibroblasts (CAFs), where it manifests as an increase in aerobic glycolysis and a hypofunctional tricarboxylic acid (TCA) cycle. CAFs would release lactate and ketone bodies into the interstitium and be captured by tumor cells. Once inside the cells, these molecules feed and enhance TCA activity. Likewise, the activation of autophagy in CAFs releases a large number of amino acids into the interstitium, which would be captured by the cells of the tumoral parenchyma for use in the anabolic synthesis of protein. Catabolic reactions predominate in the stromal CAFs, favoring the preponderance of anabolism in the tumor cells. This hypothesis is known as the reverse Warburg effect or the coupling model [54].

The acidity and hypoxia of the interstitium and the relative deficit of glucose and amino acids induce functional changes in the different cellular subpopulations of the interstitium, including myofibroblasts, endothelial cells, T lymphocytes, and macrophages, among others. All these functional changes are mainly aimed at promoting tumor growth. In this sense, immune cells favor tissue growth through immunosuppressive changes [3].

### 2.3. Metabolic Similarities between the Physiological Tumor Regenerative Phase and Tumor Growth

The processes of tissue injury repair, such as wound healing, are self-limited. Once the repair has been effected, the parenchymal cells stop proliferating, and the stroma composition normalizes. However, in cancer, the process is not self-limited and never ceases. As occurs in tumors, ROS, particularly H_2_O_2_ (hydrogen peroxide), are essential for activating the stroma to initiate wound healing. The inhospitable conditions faced by tumor stromal cells are similar to those present in wounds, where there is strong glucose uptake, followed by lactate secretion by parenchymal cells [55] and a high level of glutamine intake [56,57]. In addition, hypoxia is also temporary before the onset of angiogenesis in wounds [58].

Glucose consumption by the parenchyma in tumors and wounds causes glucose depletion in the interstitium, with consequences for different cell lines reminiscent of what happens in the regenerative phase of wound repair. First, the proinflammatory activity of M1 macrophages and effector T cells is limited. In parallel, it favors the M2 phenotype of macrophages and the secretion of TGFβ by T cells, which activate the differentiation and function of fibroblasts, transforming them into myofibroblasts in wounds and cancer associated-fibroblasts (CAFs) in tumors [59,60]. Myofibroblasts have an aerobic glycolysis metabolism, and their proliferation and extracellular matrix formation are very sensitive to the lack of glucose [61,62]. It has been proposed that the shortage of glucose in the interstitium of wounds is a self-limiting system of excess fibrosis in healing that could prompt functional deterioration. Additionally, the transition from the inflammatory to the regenerative phase occurs by depleting glucose in the interstitium [63].

The role of lactate in the tumor interstitium is reminiscent of that in the regenerative phase of inflammation. As in tumors, the lactate concentration in the wound interstitium is very high [64]. Lactate promotes tissue regeneration and wound healing and is a chemoattractant of fibroblastic-type mesenchymal cells, favoring collagen deposition [55,65,66]. Evidence for this comes from the observations that exogenous lactate stimulates neovascularization [67] and wound healing in limbs [68]. Direct treatment of wounds with lactate stimulates the production of VEGF (Vascular Endothelial Growth Factor) by macrophages and thereby promotes angiogenesis [55,69]. Hypoxia also contributes to macrophage immunosuppression. Indeed, the observation that the physiological process of wound healing is enhanced in anaerobiosis by lactate production was described many years ago [70]. Activation of autophagy in interstitial myofibroblasts by oxidative stress occurs under physiological conditions and, as in cancer, involves the stabilization of HIF1α (Hypoxia-Inducible Factor 1α) [36,71,72,73].

Aerobic glycolysis is also a feature of M1 macrophages induced by DAMPs and bacterial endotoxin during inflammation [74,75]. Indeed, aerobic glycolysis promotes the proinflammatory M1 state in macrophages, which is typical of the first stage of inflammation [76]. This effect could be mediated by pyruvate dehydrogenase kinase, the enzyme that limits the entry of pyruvate into the tricarboxylic acid cycle (TCA) [77].

The ability of the M1 phenotype of macrophages to change to an M2 phenotype is promoted during wound healing, in which amino acid depletion limits the inflammatory phase and induces the repair phase. The local lack of tryptophan also contributes to restricting the inflammation due to the high activity of the IDO-1 (indoleamine 2, 3-dioxygenase 1) enzyme of macrophages and fibroblasts, which rapidly transforms l-tryptophan to kynurenine promoting the differentiation of T cells and tissue repair [78,79].

**Figure 1:**
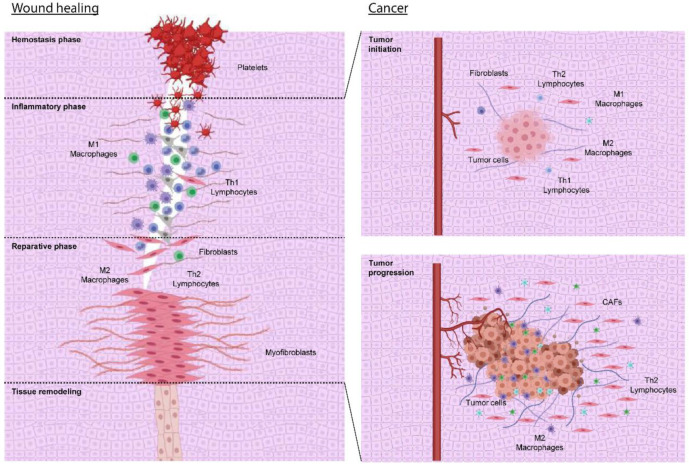
There is a parallel between wound healing and tumor growth. Wound healing consists of four consecutive phases (see text). A TH1 (T helper 1 cell)/M1 (M1-type macrophage) response predominates in the inflammatory phase, whose function is to eliminate necrotic debris and microorganisms. Once the tissue lesion is sterilized, the reparative phase begins. An anti-inflammatory and immunosuppressive TH2 (T helper 2 cells)/M2 (M2-type macrophage) activity predominates in this stage, favoring fibroblast activation and transformation into myofibroblasts. The metabolic changes of the lesion favor the change from TH1/M1 activity to TH2/M2 response. All this is very similar to what happens in tumors, where, in the initial stage, some inflammatory activity might be responsible for eliminating tumor cells that are sufficiently different from the antigenic point of view (immunoediting). A chronic regenerative phase then begins, in which TH2/M2 activity predominates and does not end because the tumor parenchyma does not stop sending signals to the surrounding stroma that allow tumor growth. Figure created using BioRender.

In conclusion, cancer has its physiological counterparts in injury repair and wound healing processes, which involve very similar molecular and cellular mechanisms. The difference is that the pseudo-repair process of the tumor is constantly active because of the unceasing demand for growth by the cancer parenchymal cells. This behavior is reminiscent of the molecular and cellular processes in chronic wounds. Ultimately, the process recapitulates the regenerative phase of wound repair. Therefore, cancer may be conceived of as a chronic regenerative phase (Figure 1). Indeed, it has been proposed that the hallmarks of cancer are also the hallmarks of wound healing [4].

## 3. Towards an Evolutionary Integration: Susceptibility and Cancer Progression as a Consequence of Antagonistic Pleiotropy of Genes That Participate in the Repair Phase of Tissue Injuries

As Theodosius Dobzhansky famously proclaimed, “Nothing in Biology makes sense except in the light of Evolution” [6]. In the present context, therefore, we should expect to find that the molecular and cellular mechanisms involved in repairing lesions have evolved towards greater efficacy during their evolution. The molecular and cellular mechanisms of the inflammatory and regenerative phases were subjected to selection pressure; in the former case because, among other reasons, the regenerative phase does not begin until the inflammatory phase has finished removing microorganisms from the wounds. Thus, the evolution of these cellular and molecular processes to fight infections is probably one of the main factors involved in their architecture. In short, the molecular and cellular mechanisms for fighting infection and repairing injuries have improved and co-evolved with infectious microorganisms that contaminate lesions and may eventually progress to septicemia. Tumors use similar repair mechanisms to ensure their growth. Antagonistic pleiotropy is determined by genes associated with advantageous traits for the development of the species, usually during the reproductive period; however, it has harmful effects in later stages of life [80,81].

Therefore, it is possible to consider cancer as a process derived from the antagonistic pleiotropy of genes whose function has been selected during evolution to participate in the efficient elimination of microorganisms and tissue repair.

As already mentioned, microorganisms must be eradicated during the inflammatory phase to ensure that the regenerative phase of wound healing begins. Therefore, there must have been significant selection pressure to remove microbes and prevent chronic wounds effectively [82]. Indeed, host genetics influences the elimination and colonization of microorganisms in wounds [83]. There are genetic loci associated with having or not having skin infections that encompass candidate genes associated with the innate immune system [84,85]. Host genetics influence the bacterial flora of wounds and the degree of difficulty in achieving wound healing. For example, they determine the bacterial composition of chronic ulcers and normal tissues, such as *Staphylococcus aureus* colonization in normal individuals [86,87]. Also, immune responses for the same microorganisms differ between individuals [88].

However, as we will see, a more complex scenario emerges from evidence suggesting that the TH1/M1 inflammatory phase is also necessary for proper subsequent tissue regeneration, even in the absence of infection. Moreover, there is also evidence that the TH2 response, which is involved in the regenerative phase, reacts to infections by helminths and from insect bites, which have been crucial in mammalian evolution.

### 3.1. The Inflammatory Phase TH1/M1 Influences Correct Tissue Injury Repair

Acute inflammation is triggered by molecules derived from tissue damage or DAMPs, such as ATP, uric acid, fibrin, HMGB1 (high mobility group box 1) protein, and heat shock proteins. Inflammation can also be activated by molecules derived from pathogens, such as lipopolysaccharides, lipoteichoic acid, mannose from the bacterial wall, and viral nucleic acids, which are collectively referred to as PAMPs (pathogen-associated molecular patterns) [89,90,91]. These molecules are picked out by pattern-recognizing receptors (PRRs), which are present in immune (and other) cells, thereby activating inflammation. The PRRs can be soluble, membrane-bound, or located within the cytoplasm.

The inflammasome is a protein complex formed by: (i) a cytosolic sensor, the PRR; (ii) an adapter molecule called ASC (Apoptosis-associated Speck-like protein containing a Caspase recruitment domain), which has a domain that interacts with the PRR, and, as indicated, another domain that recruits and activates (iii) caspase 1, which activates IL-18 and IL-1β, and gasdermin D. The latter polymerizes, generating pores in the membrane causing pyroptosis, a caspase 1-dependent cell death accompanied by the release of the indicated cytokines, IL-1 β and IL-18 [92]. One of the best-characterized inflammasomes encompasses the NLRP3 (NLR family pyrin domain containing 3) receptor, previously called NALP3 (NACHT, LRR, and PYD domains-containing protein 3) [93,94,95,96].

Despite their preferentially destructive activity, the inflammation phase and the inflammasome contribute to the successful regeneration of tissue lesions. Mice deficient in NLRP3 and caspase 1 not only have a less potent inflammatory phase (producing smaller quantities of cytokines, such as IL-1β and TNFα (Tumor Necrosis Factor α), and accumulating fewer inflammatory cells, such as neutrophils and macrophages) but also experience a delay in wounding repair that is alleviated by treatment with IL-1β [97]. Furthermore, NLPR3 and ASC knockout mice both exhibit weaker inflammatory activity and delayed wound repair, although these improve when ATP, a ligand of NLPR3, is added [98]. *Nlpr3* knockout mice also have defective liver regeneration [99], making the inflammasome necessary for both the inflammatory and regenerative processes. These effects could arise because inflammatory cytokines stabilize factors with pro-regenerative activity, such as bFGF (Fibroblast Growth Factor)/FGF2, which are essential for wound repair and cytoprotection [100].

Since the primary function of the inflammatory phase is to eliminate microorganisms, mainly bacteria and fungi, from the wounds, it is not surprising that the inflammatory phase co-evolved with the ability of microorganisms to invade the body. In this way, more molecular tools of increasing effectiveness emerged to repel the microbial attack, and bacteria also co-evolved to infect hosts, similarly to how microbes respond to antibiotics.

### 3.2. The TH2 and M2 Responses Also Control Some Infections, Such as Those Arising from Helminths and Insect Bites

Helminths and insect bites are two major inducers of the TH2 response [101,102]. The most significant effect of helminth attacks is tissue destruction once they invade the body. It has been proposed that the response to helminth infection is an adaptation of the tissue to being damaged [103] and that the best strategy for the body is to tolerate them to a degree and regenerate the tissue [104,105]. This has been described in other species, even those that are phylogenetically very distinct from humans, as is the case with *Salmonidae* [106].

At the same time, the TH2 pathway facilitates other means of attacking helminths, such as IgE production and eosinophilia. Remarkably, the response to helminths may have evolved because a TH1 response to helminths, which are relatively large animals, would produce excessive tissue damage [103]. Nevertheless, coevolution with helminth infections may shape the TH2 response. This would have significant implications for the physiological repair of tissue damage and cancer, given the role of the TH2 response in tumor development.

### 3.3. Cancer May Be a Phenomenon of Antagonistic Pleiotropy by Genes Participating in the Local Immune Response and Wound Healing

In biological or genuine pleiotropy, one or more genetic variants are associated with two phenotypes that are not pathogenically related to each other. In other words, one phenotype is not causally related to another and so is not an intermediate phenotype [107,108]. By contrast, the concept of mediated pleiotropy involves genetic variants, each of which is linked to two phenotypes because one of them is causally related (i.e., it is an intermediate phenotype) to the second (which can be a complex phenotype) [109]. Antagonistic pleiotropy involves genes that are associated with favorable phenotypes for the development of the species in particular environmental conditions and that allow its evolution, usually in reproductive phases, although it has deleterious effects in later stages of life [80,81]. It is likely that genetic variants related to metabolism and the changes they induce in immune activity are related to more effective wound repair, but in later stages in life also bestow greater tumor susceptibility or more efficient tumor growth. For example, *Zbp-1*, essential for the response to viruses, has been related to a higher susceptibility to breast cancer in a mouse model [110].

For all the above reasons, cancer can be considered a process derived from the antagonistic pleiotropy of genes whose function has been selected during evolution to participate in efficient tissue repair. This implies that there is selection pressure favoring an efficient regenerative phase and TH2/M2 activity. However, there will also be selection pressure on the inflammatory phase and TH1/M1 function for the aforementioned reasons. First, the regenerative phase does not begin until the contaminant microorganisms have been eliminated, which implies coevolution between microbes and their host’s ability to eliminate them. Thus, an effective inflammatory TH1/M1 response would have evolved to destroy microorganisms. Second, the inflammatory phase participates in a subsequent efficient regenerative phase. Apart from this, it is also possible that there has been additional selection pressure on the regenerative stage, or, more specifically, on the TH2 and M2 immune response, through their action against helminth and insect bite-borne infections.

In conclusion, these molecular and cellular interactions have developed during metazoan evolution to enable the correct repair of tissue lesions. An inflammatory phase precedes tissue repair to eliminate the infection by microorganisms. The inflammatory phase ends once the wound has been sterilized, after which the reparative phase begins. There must have been selection pressure on both processes for an effective inflammatory phase to have evolved, followed by an efficient regenerative stage. Selection in the inflammatory phase required the coevolution of the mechanisms of host inflammation with those of the infection of the different microbes in their attempt to evade the host defense mechanisms. However, the molecular and cellular processes responsible for correct tissue repair also operate in effective tumor growth. On the other hand, genetic variants possibly associated with tumor susceptibility and development may simultaneously participate in properly eliminating microbes that contaminate the lesions and correct tissue repair. Therefore, cancer could be considered a phenomenon of antagonistic pleiotropy involving the genes that help restore tissue lesions. It is possible that the networks of genes involved in a more efficient regenerative phase also determine a “more efficient” endless regeneration phase during the tumor process. Indeed, many genetic variants involved in metabolism, immune response, and inflammation have also been identified as tumor-susceptibility genes (Table 1).

## 4. Parallels between Embryogenesis and Cancer

We have seen the molecular and cellular metabolic changes that occur in the local growth of the tumors and their similitude with wound healing. However, two essential characteristics of cancer would find their physiological correlate in embryonic development. On the one hand, in embryos, there is a proliferation of immature cells. Similarly, in tumors, there is a continuous proliferation of the tumor parenchyma, characteristically accompanied by a variable blockade of cell differentiation and even an aberrant differentiation [143]. Also, tumor cells can migrate and spread at a distance, a process that marks the prognosis of the disease. Although cell migration is present in the phase of reepithelialization of wounds, migration is already evident in the embryonic stage of gastrulation, and its mechanisms are shared with those of tumor dissemination [144,145].

The resemblance between embryogenesis and tumor development was already indicated by Virchow in 1859 [146] and then by Lobstein and Récamier in 1892 [147]. Today the resemblance between carcinogenesis and embryogenesis is broadly accepted. Thus, the state of high proliferation and the mechanisms of cell cycle regulation of early embryonic cells and tumor cells would be similar. Tumor stem cells and embryonic stem cells share many characteristics, so tumors, especially those with a poor degree of differentiation (such as triple-negative basal breast cancer or glioblastoma), express similar gene signatures to those of embryonic stem cells [143]. In addition, tumors with an expression of gene signatures similar to embryonic ones have worse evolution and prognosis [148]. Embryonic carcinoma cells and embryonic stem cells express similar levels of the same groups of genes [149].

The origin of an endless proliferation of the tumor has its origin in mutations that affect protooncogenes that also participate in tissue proliferation and repair. These protooncogenes often participate in embryogenesis and induce epigenetic and transcription programs that mimic those present in the embryo [150,151,152]. This explains that many genes expressed in embryonic cells, necessary for their proliferation and survival, would also be expressed in tumor cells.

There are also similarities between embryonic and tumor metabolisms [153], which are also present in wound repair. So the pressure of evolutionary selection might have also been exerted at the embryonic level and improved the metabolic changes that allow tissue growth. Certainly, aerobic glycolysis is not only a characteristic of tumors but of proliferating tissues [154], and it is already present in the embryo at the preimplantation stage [155]. Similar to what is seen in tumor growth and wound repair, an increase in glucose consumption is observed in embryos [156,157,158,159]. In addition, the blastocyst also releases large amounts of lactate, even in the presence of oxygen [156,160,161]. In both early embryos and tumors, the pentose phosphate pathway would be enhanced, which would generate large amounts of ribose necessary for the synthesis of nucleic acids and NADPH [154].

The need for glutamine is also increased in the embryo [162,163,164,165]. In embryos from different species studied, glutamine disappears rapidly from the culture medium [166,167,168]. The behavior concerning other amino acids would also be similar between embryos and tumors [169]. In short, tissue growth processes, both pathological, such as cancer, and physiological, such as wound repair, activate metabolic pathways typical of tissues with high proliferation already present in the early stages of the embryo. Therefore, in such early stages, a selection pressure could have been exerted that maintains the embryo’s viability as a proliferating tissue. Subsequently, these mechanisms would be reactivated and present in the adult’s tissue growth processes.

Therefore, possibly part of the cellular and molecular metabolic mechanisms involved in tumor growth and wound repair would have their evolutionary origins in phases as early as the embryo’s preimplantation stage. However, local tumor growth involves stromal activation with the participation of inflammatory cells, which are also present in the repair of adult tissues. In the embryo, the repair of wounds is different from that of the adult because it is not accompanied by inflammation [170,171], as it happens in postnatal life in tissue repair and tumor growth. Therefore, the metabolic, proliferation, and differentiation pathways accompanying tumor growth (and tissue repair) have already been subject to selection pressure from embryonic stages. However, the postnatal stage has outlined stromal activation with the inflammation accompanying tissue repair, whose mechanisms are also used in tumor growth. 

A partly different context is the fetal period, where inflammatory cells are already present. In viviparous vertebrates, the fetus is the developmental stage between the embryonic phase and the moment of birth. In humans, the embryonic phase lasts eight weeks from the moment of fertilization, and from the beginning of the ninth week begins the fetal stage that lasts until birth. During the fetal period, new organs and tissues are no longer formed, but the maturation of existing ones occurs.

In the fetal liver, activated stromal cells similar to those that appear in tumors and wound repair have been identified. Therefore, it has been proposed that there would be a fetal reprogramming of the stroma in adult tissue growth [172]. Thus, in the fetal liver have been identified macrophages comparable to tumor-associated macrophages (TAMs), specific endothelial cells, and myofibroblasts similar to CAFs. These same cells, with their specific markers, have also been detected in hepatocarcinoma and the cirrhotic liver, where regeneration is activated [172]. These activated cells in the fetal liver probably reflect this organ’s enormous regeneration capacity, which is unique even in adults [173,174]. Moreover, major extramedullary hematopoiesis occurs in the fetal liver [175]. Thus, these macrophages and fibroblast cells in the fetal liver likely represent the stromal activation in any growing tissue, whether normal or pathological.

Overall, the behavior of wound repair in early gestation and the fetal stage is somewhat different from that of the adult [176]. Wound repair is carried out in the embryo and the early fetal stages without forming a fibrous scar [177], which has been confirmed in fetuses of various animals and humans [176,178]. Moreover, the ability to heal without fibrosis in mammalian fetuses depends on age [179,180]. Indeed, in skin wounds in human fetuses, fibrous scar takes place from the 24th week of gestation and in mice from day 18.5 (the average gestation period for mice is 20 days) [181,182,183], although it may depend on the size of the tissue lesion [180].

The lower fibrosis observed in fetal wounds is due to poorer inflammatory response. The inflammatory reaction involves the recruitment of immune cells that produce and release mediators of inflammation, such as growth factors and cytokines that recruit more inflammatory cells. Platelets are the first cells involved in the inflammatory reaction. These are activated and aggregated and form the hemostatic plug. Platelets release PDGF, IL1 and 6, and TGF-β into the microenvironment of the lesion. Interestingly, it has been proven that fetal platelets aggregate with more difficulty, which is attributed to the high levels of hyaluronic acid in the fetal matrix. This reduces neutrophils, macrophages, and lymphocyte infiltration into wounds early in gestation [184]. A lower number of macrophages and T lymphocytes is also observed, as well as cytokine activity. Indeed, in fetal skin, there is a reduction of chemoattractant cytokines such as CCL17 (Chemokine (C-C motif) Ligand 17), CCL21, and CCL27 [185]. Furthermore, significant TGF-β activity has been detected with its potent anti-inflammatory properties that, however, do not translate into an increase in fibrosis in this context [186].

The extensive stromal fibrosis in many tumor types would indicate that their tissue repair response more closely resembles a postnatal one. However, probably the pressure of evolutionary selection on tissue growth mechanisms already began in the embryonic and fetal periods because all those processes allow individuals to reach the reproductive stage. Indeed, the selection pressure is exerted on the embryo to reach the fetal phase and, in the latter, to be born and reach the postnatal stage. However, in the postnatal stage, intense environmental pressure must be overcome to reach the reproductive stage. Within this pressure selection to survive, there would be to develop correct wound repair mechanisms to avoid infections associated with them, to permit reaching the reproductive period. At this stage, postnatal tissue repairing generates much more fibrosis with stromal activation, which is common with tumor growth [187].

## 5. Towards Systems Biology Integration: Susceptibility to Cancer as a Consequence of Genetic Determinants of Shared Intermediate Phenotypes in the Wound Repair Process

### 5.1. The Problem of Missing Heritability in Complex Traits

Sporadic cancer growth and normal tissue repair are complex traits [188,189]. Both exhibit variations in phenotypic or clinical presentation between individuals [190]. Differences in susceptibility, evolution, and response to cancer therapy between patients have been identified [191]. Differences also exist in the repair capacity between individuals at the physiological and pathological levels. This gives rise to diseases related to defects in tissue repair and wound healing [192].

Phenotypic variation between individuals with complex traits such as cancer and tissue repair is influenced by the environment and by a polygenic, quantitatively inherited component involving many small-effect genes that interact with each other and the environment. Moreover, the polygenic part of complex traits is also present in Mendelian phenotypes, such as hereditary cancer, where the polygenic component modifies the activity of the primary driver gene. This explains the clinical heterogeneity of hereditary cancer within the same family [193].

The phenotypic variance due to the polygenic component is called heritability [194,195]. The polygenic element varies between individuals because they possess different allelic variants. These allelic variants are translated into DNA sequence changes or DSVs (DNA Sequence Variants) [196].

It is possible to estimate the amount of the phenotypic variance of a complex trait due to genetics (the expected heritability) and then calculate the amount of explained heritability from the identified DSVs associated with it. However, there is a large discrepancy between the two measures, such that, in many complex traits, identified DSVs explain only 5–10% of the expected heritability. The difference between the two heritabilities is known as missing heritability and is one of the main characteristics of complex traits, including sporadic cancer [8,197] and tissue repair [198,199]. The best way of identifying the missing polygenic component of complex traits continues to be debated [8,200,201,202].

### 5.2. Complex Phenotypes Such as Cancer and Tissue Repair Arise from Multiple Intermediate Phenotypes

Many important pathophysiological diseases, such as sporadic cancer, diabetes mellitus, ischemic heart disease, autoimmune diseases, and physiological processes, such as height, intelligence, and wound repair, are complex phenotypes. These traits result from another series of second-order or intermediate processes or phenotypes causally related to the complex trait and involved in its pathogenesis. These intermediate phenotypes are found at the systemic, tissue, cellular, or molecular level. Furthermore, the levels and compartments interact with each other and facilitate the homeostasis of the organism.

On the other hand, in many cases, the pathophysiological, cellular, and molecular components of one or more of these levels will be simultaneously involved in the pathogenesis of several complex traits, thereby influencing their concurrent expression. This explains the epidemiological association between complex diseases arising from the existence of common pathogenic processes. As an example, this would be the association of autoimmune processes with cancer, or cancer and obesity; inflammation and obesity, or cancer and aging. In a broader sense, and considering the similarities between pathogenic processes, an attempt has been made to group various diseases by family, thereby redefining the taxonomy of disease and giving rise to the concept of the diseasome [203,204]. It is very likely that extending this taxonomy to encompass physiological and pathological processes will reveal a clear connection between cancer and tissue repair.

There are various explanations for the missing heritability [205]. We have proposed that it is due to the phenotypic architecture of the complex traits, which comprises a multitude of intermediate phenotypes at the systemic, tissue, cellular, and molecular levels [7]. It could be due to genes that exert their influence at the intermediate phenotypic level, but they would not be powerful enough to be detected at the level of the primary complex phenotype [7,206], in whose pathogenesis they participate. This possibility is consistent with the heritability being missing because differences between many common variants cannot be significantly associated with the complex phenotype [207,208,209,210]. Therefore, we hypothesized that genes lacking sufficient strength to be detected at the main trait level as examples of mediated pleiotropy of intermediate phenotypes account for a substantial proportion of the missing heritability of this complex trait [7].

Attributing missing heritability to the genetic determinants that influence networks of intermediate phenotypes involved in the pathogenesis of a complex trait disease requires thousands of genes acting on its variance and susceptibility [7]. Indeed, it was predicted that common small-effect genes affecting a complex trait might be located throughout much of the genome. For example, more than 71% of 1-Mb genomic regions could contribute to the heritability of schizophrenia [208], and thousands of expression quantitative trait loci (eQTLs) may control blood gene expression [211]. Furthermore, mathematical models predict that between 0.1 and 1% of SNPs have a causal effect on most diseases studied [212]. These observations align with the exciting omnigenic model that purports to explain missing heritability [213,214]. However, one of the difficulties is identifying genetic markers that predict the susceptibility of complex trait diseases. Efforts have been made to develop genome-wide polygenic scores based on thousands of genetic variants [212].

Tissue repair and cancer are indisputably complex traits. They share many intermediate phenotypes involved in their pathogenesis that function in inflammatory and regenerative immune responses and metabolic changes [5,15,188]. For example, the different evolution of the same type of cancer between individuals and the same genetic driver could be determined by differences in the degree of CAF activity [213,215,216], or in components of the reparative phase, such as M2 macrophages and TH2 lymphocytes [217]. These pathogenic components also participate in tissue repair and wound healing [218]. For example, a defect in tumor development such as that seen with SNAI2 (Snail family transcriptional repressor 2) [219,220] also translates into a defect in lesion repair [221,222,223], and *Snai2*-deficient CAFs have a lower capacity for producing some important cytokines in these processes [220].

### 5.3. Identifying Genetic Determinants of Cancer Susceptibility and Progression through Intermediate Phenotypes Shared with Tissue Repair

Identifying genetic variants associated with a complex phenotype through their association with intermediate phenotypes is not a new concept [224]. In the context of diseases of complex genesis, the approach has been most enthusiastically taken up in the field of psychiatric genetics [224], in which the term endophenotype, first coined by Gottesman and Shields [225], is understood to be equivalent to that of the intermediate phenotype. The rationale is that intermediate phenotypes associated with the disease are pathogenically related to it and represent more elementary phenotypes. Intermediate phenotypes have been used successfully to identify genetic determinants of complex psychiatric and non-psychiatric disorders [226,227,228,229,230,231,232].

**Figure 2:**
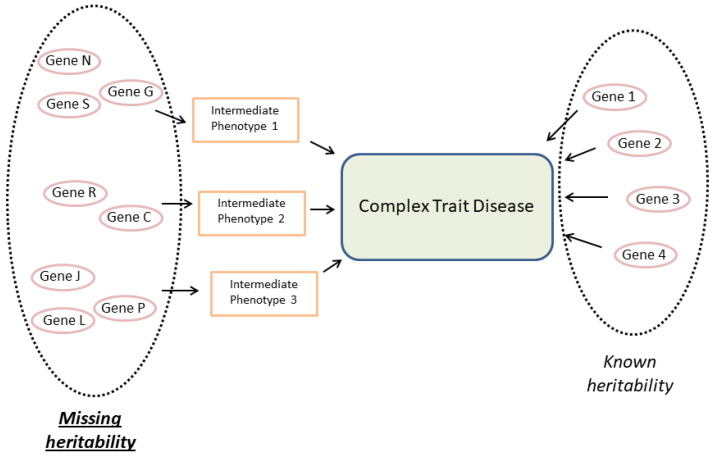
Complex traits have a polygenic component associated with the phenotypic variation that can be directly detected as known heritability. Many intermediate phenotypes are also involved in the pathogenesis of a complex trait. These intermediate phenotypes have their heritability, formed by genetic determinants that contribute to their phenotypic variation among individuals (in the figure, genetic determinants are quantitative trait loci (QTLs)). Most genetic components associated with the phenotypic variability of complex traits cannot be detected, and so comprise the missing heritability. We proposed that missing heritability could be explained by the genetic determinants associated with the heritability of intermediate phenotypes and that these elements do not have a large enough effect of being capable of detection at the level of the complex trait.

Genetic components associated with intermediate phenotypes may, in turn, be related to complex traits. Identifying them would make it possible to apportion part of the missing heritability and would improve our knowledge of the different degrees of susceptibility and patterns of evolution between individuals [7] (Figure 2). Indeed, some complex traits, such as diseases of complex genesis, are known to share common pathogenic mechanisms and even genetic variants that lead them to share a greater susceptibility to these processes. In other words, the same genetic variant can be associated with more than one trait. This phenomenon has been called cross-phenotype association [233]. In this sense, genetic variants that are simultaneously associated with tumor development and tissue repair have already been described (Table 1). This would also be the case for many genes linked to intermediate phenotypes shared by cancer and the wound-repair process [234]. The SNAI2 deficit is an example of a cross-phenotype association [220,221,222,223]. The immune and metabolic-endocrine systems and overall organ physiology influence both wound healing and tumor growth. Consequently, the possibility cannot be ruled out that these wound-healing- and cancer-related intermediate phenotypes are local and systemic. For example, changes in liver signaling pathways associated with tumor evolution [7,206] and a series of systemic metabolites related to it [7,110,206] have been described.

In conclusion, tissue injury repair and tumor growth may share many intermediate cellular and molecular phenotypes in a Systems Biology structure. Both processes are complex traits. As such, the variation in their phenotypic presentation between individuals depends on the environmental influence and its interaction with a polygenic component. In complex traits, most of this polygenic component cannot be detected and has to be ascribed to missing heritability. However, it is possible to identify genetic determinants of different susceptibility and tumor evolution by finding the genes associated with the variability of intermediate phenotypes responsible for the heterogeneity of the complex trait. It might even be possible to predict the different patterns of tumor evolution from the heterogeneous behavior of intermediate phenotypes in tissue repair.

## 6. Concluding Remarks

Cancer is the consequence of many molecular and cellular intermediate phenotypes whose physiological correlate is found in the equivalent processes involved in tissue repair. These molecular and cellular processes have arisen during the biological evolution of organisms under the pressure of selection to overcome external aggressions, mainly accidents and microorganisms. Therefore, some of the differences in susceptibility and tumor evolution between individuals may be explained by differences in the behavior of these intermediate phenotypes. On the other hand, this difference in the behavior of the intermediate molecular and cellular phenotypes is, in turn, determined by the genetic variation between individuals. However, it is difficult to detect any significant association of these genetic variants with tumor phenotypic variation because their small effect does not significantly affect the level of the main complex trait and is part of its missing heritability [7]. Therefore, we may be at the limit of the ability of strategies such as genome-wide association studies (GWAS) to identify genes responsible for the susceptibility of complex traits such as cancer. New RNA-Seq strategies can help identify an additional part of missing heritability [235], especially concerning splicing variants, noncoding regulatory zones, and lncRNAs (long non-coding RNAs). However, we will always be limited by the small individual effect these genetic variants have on the complex trait that will not permit their localization. For this reason, it would probably not be easy to go further, even with the application of new techniques, such as new-generation RNA-seq.

Like any complex trait, cancer is composed of more simple intermediate phenotypes at the molecular and cellular levels. However, these intermediate molecular and cellular phenotypes directly influence other intermediate phenotypes that are complex traits to a greater or lesser extent. For example, in the case of cancer, intermediate phenotypes, which in part are complex traits, are angiogenesis, apoptosis, proliferation, or dissemination. It is difficult to dissect a complex trait’s molecular and cellular architecture thoroughly and identify the genetic variants involved in it. However, it is possible to use intermediate phenotypes as surrogates of the complex trait, and it is possible to identify genetic determinants that form part of the missing heritability but could significantly contribute to the variability of the complex trait. In doing so, including the effect of different genetic variants in mathematical models featuring those genetic markers directly associated with the main trait could help reveal the effect of genetic determinants on complex trait variability. Combining multiple genetic variants that explain complex intermediate phenotypes could also help explain tumor susceptibility and bring us closer to providing more individualized medicine.

## Figures and Tables

**Table 1:** Summary of genes implicated in cancer and metabolism, infection, or the immune system.

Cancer	Gene	SNP	Molecular Function	Reference
Lung cancer	*JAML* or *AMICA1*	rs1056562	It encodes a protein of the leukocyte plasma membrane that controls its migration and activation through interaction with CXADR, a plasma membrane receptor found on adjacent epithelial and endothelial cells. The interaction between the two receptors mediates the activation of gamma-delta T cells, a subpopulation of T-cells involved in tissue homeostasis and repair.	[111]
	*BLK*	rs1600249	It encodes a non-receptor tyrosine kinase involved in B-lymphocyte development, differentiation, and signaling.	[112]
	*IL2RB*	rs3218339	It encodes the beta subunit of the interleukin 2 receptor, whose ligand plays an essential role in the proliferation of T and B lymphocytes.	[113]
	*PDCD1LG2*	rs7854413	Protein whose interaction with PDCD1 inhibits T-cell proliferation by disrupting cell cycle progression and cytokine secretion.	[114]
	*SYK*	rs10761395	Its encoded protein helps couple activated immunoreceptors to diverse cellular responses such as proliferation, differentiation, and phagocytosis.	[113]
	*IDO1*	rs10108662	This enzyme catalyzes the first step in tryptophan catabolism and is thought to be active in antimicrobial and antitumor defense, immunoregulation, and antioxidant activity.	[113]
Gastric cancer	*PRKAA1*	rs10074991	It encodes a catalytic subunit of AMP-activated protein kinase (AMPK), which regulates energy metabolism in cells, thereby increasing the AMP/ATP ratio.	[115]
	*DEFB121*	rs2376549	It encodes a member of the beta subfamily of defensins, antimicrobial peptides that protect tissue and organs from infection by various microorganisms.	[116]
Esophageal cancer	*FASN*	rs17848945	It synthesizes a fatty acid synthase that catalyzes the formation of long-chain fatty acids involved in cell metabolism.	[117]
	*STING* or *TMEM173*	rs7447927	It encodes a transmembrane protein that is a major regulator of the innate response to viral and bacterial infections, activating type I interferon signaling.	[118]
Pancreatic cancer	*SUGCT*	rs17688601	It catalyzes the succinyl-CoA-dependent conversion of glutarate to glutaryl-CoA, which is involved in cell metabolism.	[119]
	*BCAR1*	rs7190458	The encoded protein acts in multiple cellular pathways, including cell motility, cell cycle control, and apoptosis. It is also involved in BCAR3-mediated inhibition of TGFB signaling.	[120]
Breast cancer	*CDCA7*	rs1550623	It induces anchorage-independent growth and clonogenicity in lymphoblasts.	[121]
	*CDKN2B-AS1*	rs62560775	It is known to recruit a polycomb repression complex (PRC2) that silences CDKN2B.	[122]
	*HNF1A*	rs2464195	It is a transcription factor required for the expression of several liver-specific genes.	[123]
Ovarian cancer	*IFNL3*	rs688187	It encodes a cytokine whose expression can be induced by viral infections.	[124]
Endometrial carcinoma	*EIF2AK4*	rs937213	It encodes a protein that responds to amino acid deprivation, glucose deprivation, and viral infection.	[125]
Laryngeal squamous cell carcinoma	*AIF1*	rs2857595	It plays a role in macrophage activation and function and promotes the proliferation of T lymphocytes.	[126]
Lymphomas	*HLA-B*	rs2523607	It participates in the presentation of exogenous antigens to the immune system.	[127]
Prostate cancer	*CDKN2B-AS1*	rs1011970	It is known to recruit a polycomb repression complex (PRC2) that silences CDKN2B.	[122]
	*RFX6*	rs339331	It regulates transcription factors involved in beta-cell maturation and function. It is also involved in glucose-stimulated insulin secretion by promoting the transcription of insulin and L-type calcium channels.	[128]
Colorectal cancer	*ZBTB20*	rs10511330	It acts as a transcriptional repressor and plays a role in many processes, including glucose homeostasis and neurogenesis.	[129]
	*SMAD7*	rs6507874	The encoded protein binds SMURF2 (E3 ubiquitin ligase) and translocates to the cytoplasm, where it interacts with TGFBR1, leading to its degradation.	[130]
	*MMP2*	rs142319636	It is a ubiquitous metalloproteinase involved in diverse functions such as remodeling the vasculature, angiogenesis, tissue repair, tumor invasion, and inflammation.	[131]
Kidney cancer	*PCSK9*	rs2495478	It plays a role in cholesterol and fatty acid metabolism.	[132]
	*TCN2*	rs2283873	It is a primary vitamin B12-binding and transport protein.	[132]
Thyroid cancer	*SMAD3*	rs2289261	Its encoded protein acts in the TGF-β signaling pathway and transmits signals from the cell surface to the nucleus, regulating gene activity and cell proliferation.	[133]
Cutaneous squamous cell carcinoma	*IRF4*	rs12203592	It plays an essential role in regulating interferons in response to viral infection and interferon-inducible genes.	[134]
	*RALY/ASIP*	rs6059655	Its encoded protein acts in the TGF-β signaling pathway and transmits signals from the cell surface to the nucleus, regulating gene activity and cell proliferation.	[134]
	*HLA-DQA1*	rs4455710	It participates in the presentation of antigens to the immune system.	[135]
Testicular germ cell cancer	*LIPG*	rs9951026	Its encoded enzyme is synthesized in endothelial cells and exhibits phospholipase and triglyceride lipase activities.	[136]
Hepatocellular carcinoma	*VEPH1*	rs2120243	Its encoded protein interacts with TGF-β receptor type-1 (TGFβR1) and inhibits the dissociation of activated SMAD2 from TGFBR1, impeding its nuclear accumulation and impairing TGF-β signaling.	[137]
	*HLA-DPA2*	rs2295119	It participates in the presentation of antigens to the immune system.	[138]
	*IFNL4*	rs8107030	The encoded protein is a cytokine that may trigger an antiviral response activating the JAK-STAT pathway, specifically upregulating some interferon-stimulated genes.	[139]
	*HLA-C*	rs1131096/rs1130838	It participates in the presentation of antigens to the immune system.	[140]
	*IL6R*	rs2228145	It encodes a subunit of the interleukin-6 receptor complex, which plays an essential role in the immune response through IL6.	[140]
	*IL7R*	rs6897932	The encoded protein is a receptor for interleukin 7, a cytokine with a critical role in V(D)J recombination during lymphocyte development.	[140]
Glioblastoma	*RHBDF1*	rs2562152	It encodes a protein involved in sleep, cell survival, proliferation, migration, and inflammation through ADAM17 protease.	[141]
Bladder cancer	*P3H2*	rs710521	It encodes prolyl 3-hydroxylase, which catalyzes the post-translational formation of 3-hydroxyproline on different types of collagen, such as COL4A1 and COL1A1.	[142]

## Data Availability

Not applicable.
